# Supplementary material for: Abnormal Morphology and Synaptogenic Signaling in Astrocytes Following Prenatal Opioid Exposure
Source: Cells. 2024 May 14;13(10):837. doi: 10.3390/cells13100837 (PMC11119541; doi:10.3390/cells13100837)
Supplement: Supplementary file 1 [file cells-13-00837-s001.zip › Figure S1 with legend.pdf]

*Figure S1*

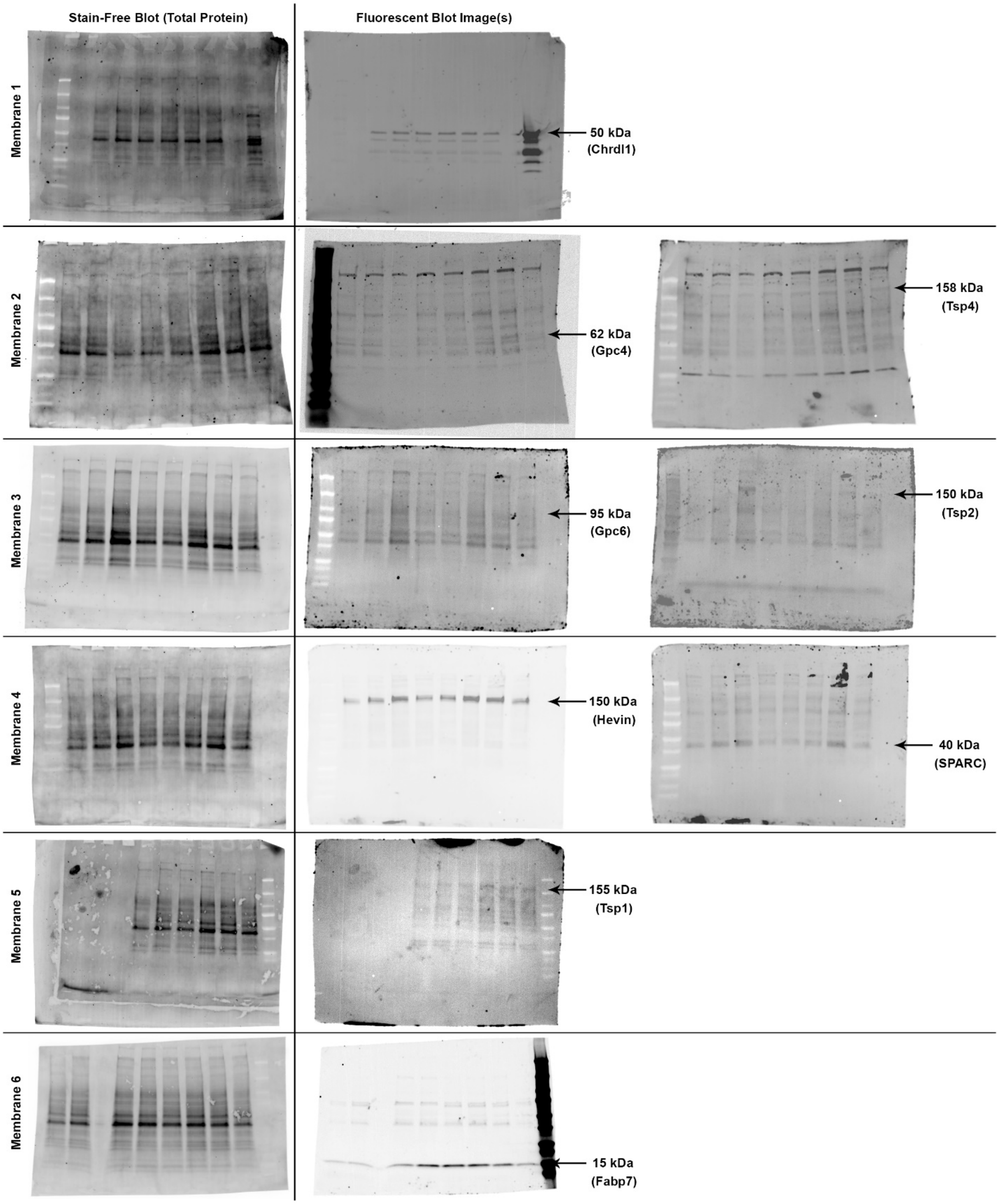


Uncropped Western blot membranes showing total protein content used for normalization along with the associated fluorescent blot images for the various primary antibodies probed.
